# Environmental stiffness restores mechanical homeostasis in vimentin-depleted cells

**DOI:** 10.1038/s41598-023-44835-8

**Published:** 2023-10-26

**Authors:** Janine Grolleman, Nicole C. A. van Engeland, Minahil Raza, Sepinoud Azimi, Vito Conte, Cecilia M. Sahlgren, Carlijn V. C. Bouten

**Affiliations:** 1 Department of Biomedical Engineering, Soft Tissue Engineering and Mechanobiology, Eindhoven University of Technology, Eindhoven, 5612AE The Netherlands; 2 Institute for Complex Molecular Systems, Eindhoven University of Technology, Eindhoven, 5600MB The Netherlands; 3 Faculty of Science and Engineering, Cell Biology, Åbobo Akademi University, 20520 Turku, Finland; 4 Faculty of Science and Engineering, Information Technology, Åbobo Akademi University, 20500 Turku, Finland; 5 Department of Information and Communication Technology, Technology, Policy and Management, Delft University of Technology, Delft, 2600GA The Netherlands; 6 Institute for Bioengineering of Catalonia, The Barcelona Institute of Science and Technology, 08036 Barcelona, Spain

**Keywords:** Intermediate filaments, Biophysics, Mechanotransduction

## Abstract

Recent experimental evidence indicates a role for the intermediate filament vimentin in regulating cellular mechanical homeostasis, but its precise contribution remains to be discovered. Mechanical homeostasis requires a balanced bi-directional interplay between the cell’s microenvironment and the cellular morphological and mechanical state—this balance being regulated via processes of mechanotransduction and mechanoresponse, commonly referred to as mechanoreciprocity. Here, we systematically analyze vimentin-expressing and vimentin-depleted cells in a swatch of in vitro cellular microenvironments varying in stiffness and/or ECM density. We find that vimentin-expressing cells maintain mechanical homeostasis by adapting cellular morphology and mechanics to micromechanical changes in the microenvironment. However, vimentin-depleted cells lose this mechanoresponse ability on short timescales, only to reacquire it on longer time scales. Indeed, we find that the morphology and mechanics of vimentin-depleted cell in stiffened microenvironmental conditions can get restored to the homeostatic levels of vimentin-expressing cells. Additionally, we observed vimentin-depleted cells increasing collagen matrix synthesis and its crosslinking, a phenomenon which is known to increase matrix stiffness, and which we now hypothesize to be a cellular compensation mechanism for the loss of vimentin. Taken together, our findings provide further insight in the regulating role of intermediate filament vimentin in mediating mechanoreciprocity and mechanical homeostasis.

## Introduction

Mechanical force controls fundamental processes in health and disease^1^. For a tissue to be functional, cells within the tissue need to establish, maintain, and restore a preferred morphological and mechanical state, a phenomenon referred to as mechanical homeostasis^2^. Loss of mechanical homeostasis is associated with the onset and progression of pathologies, ranging from cardiovascular diseases such as cardiomyopathy^3^ and aneurysms^4^ to cancer^5^. The regulation of mechanical homeostasis requires fine-tuned mechanoreciprocity, which is defined as the dynamic bi-directional mechanical interplay between a cell and its microenvironment rich in extracellular matrix (ECM)^6,7^. Mechanoreciprocity requires mechanical signals from the cellular environment to be sensed by the cells and converted into biomechanical and biochemical signals in the nucleus (mechanotransduction), which in turn triggers a response from the cell at the mechanical level (mechanoresponse). This leads to adaptation of the cell’s morphological and mechanical (morpho-mechanical) state, on a timescale of seconds to hours, as well as to the synthesis and remodeling of the ECM, occurring within a timespan of days^6,8^.

The complex regulation of the continuous adaptive remodeling process of mechanoreciprocity is known to involve the cytoskeleton^9,10^. The cytoskeleton is a dynamic network composed of actin, microtubules, and intermediate filaments (IFs), and is important for transmitting mechanical signals from the ECM to the nucleus^11^. The cytoskeleton also governs the morpho-mechanical state of a cell, and extensive experimental evidence has elucidated the roles of stretch-resistant actin and compression-resistant microtubules in maintaining cellular mechanical homeostasis^12^. Yet, little is still known about the contribution of IFs to the mechanical homeostatic balance.

The IF protein vimentin is involved in many processes driven by cell mechanics such as wound healing, angiogenesis, flow-induced arterial remodeling, and closure of the ductus arteriosus (reviewed in ref^13^). Vimentin is highly expressed in mesenchymal cells, including fibroblasts, endothelial cells (ECs), and vascular smooth muscle cells (VSMCs)^14^, where it appears as a cage-like network surrounding the nucleus and is crucial in regulating nuclear morphology^15^. The vimentin network extends to the cell cortex, including protrusions, where it forms an interpenetrating network with the actin cytoskeleton^16^. Both vimentin and actin associate with focal adhesions (FAs), the protein complexes that mechanically couple the cytoskeleton to the ECM at the cell membrane^17,18^. FA dynamics is known to be controlled by vimentin^19–21^ and is highly dependent on the mechanical characteristics of the ECM, including ECM stiffness and density^22^. Recent studies have been highlighting a regulatory role for vimentin in the synthesis and remodeling of the ECM in vivo, as increased ECM production and tissue stiffening is observed in vimentin-depleted mice^23,24^. Thus, we hypothesize that vimentin regulates mechanoreciprocity, but *how* vimentin contributes to mechanical homeostasis is not known (Supplementary Fig. S1).

In this study, we systematically investigate the role of vimentin in mechanical homeostasis by quantifying how vimentin depletion affects ECM expression and the morpho-mechanical state of cells from arterial tissues cultured in a swatch of mechanical microenvironmental conditions varying in stiffness and ECM density. We find that vimentin is essential for cells to restore mechanical homeostasis on shorter timescales by initiating an adaptive mechanical response to changes in stiffness and ECM density of the cell’s microenvironment. Our data shows that vimentin depletion in cells initially disrupts their mechanoresponsive ability and then triggers an increase in their expression of ECM components and collagen crosslinkers on a longer timescale. Surprisingly, vimentin-depleted cells are able to restore their morphology and mechanics to the homeostatic levels of vimentin-expressing cells, provided that the stiffness of these cells’ microenvironment is sufficiently increased independently of ECM density.

## Results

### Vimentin regulates ECM production and remodeling

**Figure 1:**
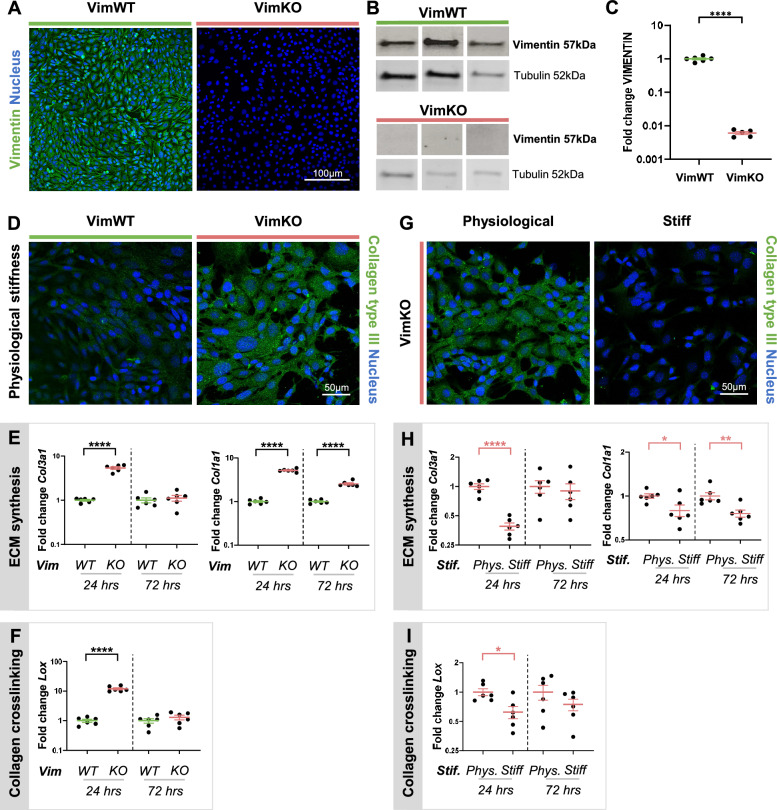
Increased substrate stiffness compensates for vimentin depletion altered ECM synthesis and crosslinking. Verification of vimentin knockout on protein level using immunofluorescence (IF) staining (**A**, N = 4) and Western Blotting (**B**, N = 3), and on gene level using q-PCR with respect to *Gapdh* expression and normalized to VimWT expression levels (**C**, N = 6). (**D**–**F**) VimWT and VimKO cells cultured on PAA gels with physiological substrate stiffness (12 kPa). Collagen type III protein expression after 72 h by IF staining (**D**, N = 6) and gene expression after 24 and 72 h by q-PCR of ECM synthesis gene *Col3a1* and *Col1a1* (**E**, N = 6), and collagen crosslinking gene *Lox* (**F**, N = 6) with respect to *Gapdh* expression and normalized to VimWT expression levels. (**G**–**I**) VimKO cells cultured on PAA gels with physiological substrate stiffness (Phys.) and on a stiff substrate (glass). Collagen type III protein expression after 72 h by IF staining (**G**, N = 6) and gene expression after 24 and 72 h of ECM synthesis genes *Col3a1* and *Col1a1* (**H**, N = 6), and collagen crosslinking gene *Lox* (**I**, N = 6). N represents the number of samples. Data is represented as mean ± SEM. An unpaired t-test was used for statistical analysis. Denotations: ****p < 0.0001; ***p < 0.001; **p < 0.01; *p < 0.05; p < 0.1.

Previous research has shown that loss of vimentin results in increased ECM production and remodeling in vivo^23,24^. To better understand the changes in ECM turnover due to loss of vimentin we cultured vimentin-wildtype (VimWT) and vimentin-knockout (VimKO) mouse embryonic fibroblasts (MEFs) on collagen type I coated polyacrylamide (PAA) hydrogel substrates having cardiovascular physiological stiffness of 12 kPa^25,26^. Vimentin knockout was verified on both protein (Fig. 1A,B and Supplementary Fig. S2) and gene level (Fig. 1C). Next, we stained VimWT and VimKO cells with an antibody against collagen type III to discriminate from the collagen type I coating. After 72 h of cell culture on the hydrogel, VimKO cells expressed a net increase in collagen type III as compared to VimWT cells (Fig. 1D), which is in line with the in vivo observations^23,24^. To refine the temporal effect and the balance in synthesis and remodeling of this finding, we analyzed the relative mRNA expression of ECM synthesis and remodeling genes in VimKO cells and VimWT cells after 24 and 72 h. The loss of vimentin significantly increased mRNA levels of *Col3a1* and *Col1a1* after 24 h of cell culture (Fig. 1E). A similar vimentin depletion related increase in ECM synthesis was observed in other vascular cell types, endothelial (Supplementary Fig. S3) and vascular smooth muscle cells (Supplementary Fig. S4), which highlight that this phenomenon is a cell generic response. In addition, we observed an increase in the expression of the collagen crosslinking gene *Lox* (Fig. 1F) and the matrix remodeling genes *Mmp2* and *Timp2* (Supplementary Fig. S5A). After 72 h of culture on the 12 kPa substrate, the increase in *Col3a1* and *Lox* normalized to the expression levels of VimWT cells, while the expression of *Col1a1*, *Mmp2* and *Timp2* remained increased. This suggests that there is an initial boost of collagen type III synthesis and collagen crosslinking, and prolonged synthesis of collagen type I and matrix remodeling.

Apart from increased ECM production, in vivo studies showed arterial stiffening as a result of vimentin depletion. To investigate the effect of changes in stiffness of the microenvironment, we cultured VimWT and VimKO cells on physiological substrate stiffness and high substrate stiffness (glass) to mimic arterial stiffening and compared the production of ECM components and regulators. For VimWT cells, we observed no significant difference in collagen type III expression on stiff substrates compared to physiological substrate stiffness (data not shown). However, contrary to VimKO cells on physiological stiffness, VimKO cells cultured on stiff substrates showed reduced collagen type III protein levels (Fig. 1G). Further analysis demonstrated a decrease in the expression of matrix genes *Col3a1, Col1a1* (Fig. 1H), *Lox* (Fig. 1I), *Mmp2,* and *Timp2* (Supplementary Fig. S5B) after 24 h of VimKO cell culture on stiff substrates compared to physiological stiffness. After 72 h of cell culture, VimKO expression levels on stiff substrates normalized to the expression levels of VimKO cells on physiological stiffness except for ECM synthesis gene *Col1a1 and* remodeling gene *Timp2*. Taken together, these data show that vimentin mediates ECM synthesis, crosslinking and remodeling depending on environmental stiffness and suggest that vimentin-depleted cells increase their extracellular matrix production unless they are cultured on substrates with increased stiffness.

### Vimentin-depleted cells adopt a differential morpho-mechanical phenotype

**Figure 2:**
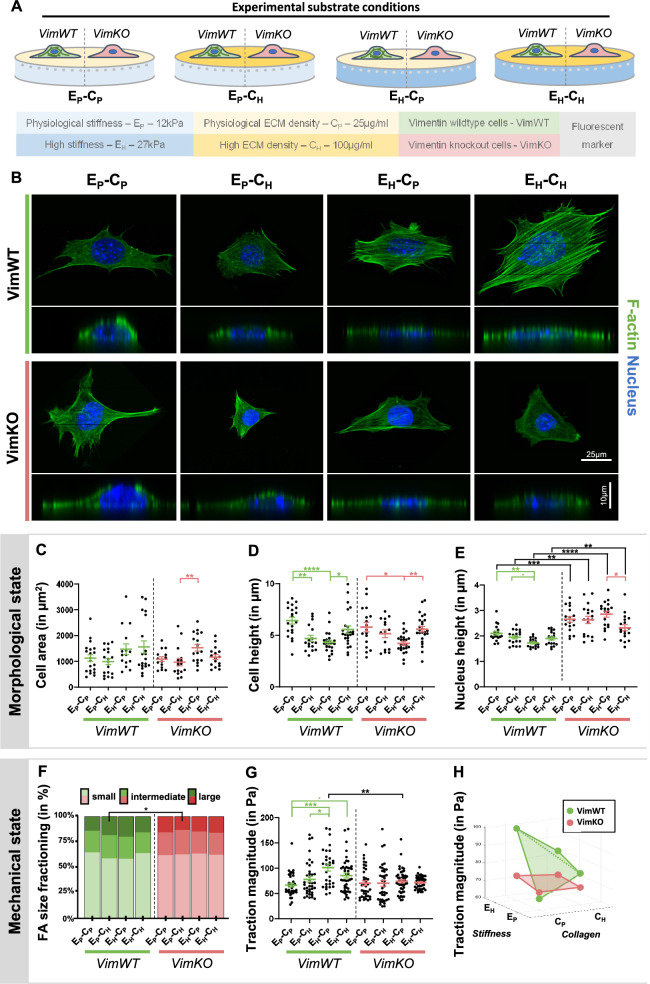
Vimentin depleted cells lose the ability to mechanically respond to alterations in the microenvironment after 24 h. (**A**) Schematic representation of VimWT and VimKO cells cultured on different substrate conditions: physiological (E_P_) vs stiff (E_H_) substrate stiffness and physiological (C_P_) vs high (C_H_) substrate ECM density. VimWT and VimKO cells were cultured on the different substrate conditions for 24 h. (**B**) Top and side confocal images of VimWT and VimKO cells cultured on different substrate conditions for 24 h stained for F-actin and DAPI (N = 6). (**C**–**E**) Morphological state quantification of cell area (**C**; N = 6, n = 15–20), cell height (**D**; N = 6, n = 15–25), and nucleus height (**E**; N = 3, n = 18). Each dot represents one cell/nucleus. (**F**–**H**) Mechanical state quantification. (**F**) FA were fractioned into small (size ≤ 0.18 μm2), intermediate (0.18˂size ≤ 0.36 μm2), and large (size˃0.36 μm2) FAs (N = 3, n = 8–14). (**G**) Traction magnitude was computed by taking the median traction value of each cell per timepoint and taking the mean over time meaning that each dot represents on cell in time (N = 6, n = 31–47). (**H**) Different representation of traction magnitude. Dots represent group mean and are connected in a bending plane for VimWT cell (in green) and a flat plane for VimKO cells (in red). N represents the number of gels, while n represents the number of cells within the quantitative analysis. Data is represented as mean ± SEM. Kruskall-Wallis followed by Dunn’s multiple comparison was used for statistical analysis between substrate conditions within VimWT cells (displayed in green) and VimKO cells (displayed in red). A Mann–Whitney test was used for statistical analysis between VimWT and VimKO cells on different substrate conditions (displayed in black). Denotations: ****p < 0.0001; ***p < 0.001; **p < 0.01; *p < 0.05; p < 0.1.

The observed changes in the ECM composition (stiffness and ECM density) prompted us to study how these mechanical alterations within the microenvironment of the cell affect the morpho-mechanical state of the cell. To this end, we cultured VimWT and VimKO cells on substrates with physiological (E_P_) or high (E_H_) substrate stiffness and with physiological (C_P_) or high (C_H_) substrate (collagen type I) ECM density (Fig. 2A) and studied the morpho-mechanical adaptation response after 24 h.

To observe differences in the morpho-mechanical state of the cell, we provide a quantitative measure of the morpho-mechanical state of the cell. Briefly, the morphological state was quantified by nuclear and cellular morphology, whereas the mechanical state was quantified by FA formation and cellular traction forces (Supplementary Fig. S6). To characterize cellular morphology, we stained for cytoskeletal protein F-actin, acquired top- and side view images (Fig. 2B), and quantified cell area and height. Both VimWT and VimKO cells showed an increasing trend in cell area to increased substrate stiffness (Fig. 2C) which is in agreement with the paradigm of increased cell spreading on stiffer substrates^27–30^. In line with this, both cell types decreased in height when cultured on hydrogels with either high substrate ECM density on physiological stiffness or high substrate stiffness with physiological ECM density, whereas combined high substrate stiffness and high substrate ECM density did not affect cell height (Fig. 2D). Next, we examined if vimentin affects nuclear morphology by acquiring top- and side view images of the nucleus (Supplementary Fig. S7A) and quantified nucleus area and height. Quantification of the area of the nucleus showed increased nuclear area in VimWT and VimKO cells on high substrate stiffness (Supplementary Fig. S7B). Additionally, a reduction in nuclear area in VimKO cells compared to VimWT cells independent of substrate was observed (Supplementary Fig. S7B). VimWT cells showed a similar but less pronounced effect of changes in the microenvironment in nuclear height compared to cellular height. Independent of the microenvironment, VimKO cells have higher nuclei compared to VimWT cells (Fig. 2E). While our data on nuclear morphology is in line with previous research showing that vimentin depletion results in rounder nuclei^15,31^, we found that cellular morphology is not affected by the presence of vimentin, most likely since vimentin is highly localized in the core of the cytoplasm and to a lesser degree at the cell periphery^16^.

As for the mechanical state of the cell we studied the formation of actin stress fibers, myosin II activity, FA formation and tractions forces^32^, which are known contributors to cellular functions including cell morphology, cell migration and ECM organization^33^. Both VimWT and VimKO cells display comparable F-actin organization when cultured on physiological stiffness independently of ECM density (Fig. 2B). However, VimWT cells formed abundant actin stress fibers when cultured on high substrate stiffness independently of ECM density, whereas VimKO cells did not exhibit comparable actin stress fibers to VimWT cells on high substrate stiffness (Fig. 2B). Besides actin organization, we assessed the effect of vimentin depletion on myosin II activity by detection of phosphorylated myosin light chain (pMLC). Previous research by Jiu and colleagues showed that VimKO cells show increased pMLC protein expression levels compared to VimWT cells^34^. Independent of the substrate, we observed a similar increase in VimKO cells in pMLC expression by visual inspection compared to VimWT cells (Supplementary Fig. S7C). Vimentin depletion also affected FA formation on the different substrates as demonstrated by quantification of the FA size based on a staining for paxillin (Supplementary Fig. S7D). Upon fractioning the FAs into small, intermediate, and large FAs within our analysis, we observed an increasing trend in FA size in VimWT cells on substrates with increased substrate ECM density on physiological stiffness and increased substrate stiffness with physiological ECM density, but no change was observed in VimKO cells (Fig. 2F). Next, we examined how these changes in the mechanical components of the cell affected cell traction forces using traction force microscopy. On physiological substrate conditions, VimWT and VimKO cells show a similar contractile behavior (Fig. 2G). While VimWT cells increased their traction magnitude to increasing substrate stiffness with physiological ECM density, VimKO cells did not adapt traction magnitude upon changes in the microenvironment (Fig. 2G). A decrease in traction magnitude was observed in VimKO cells cultured on high substrate stiffness with physiological ECM density as compared to VimWT cells (Fig. 2G). Interestingly, VimWT cells increased traction magnitude upon increased substrate ECM density on physiological substrate stiffness, whereas they decreased traction magnitude upon increased substrate ECM density on high substrate stiffness (Fig. 2H) indicating that there is an optimal substrate condition at which VimWT cells are able to exert the highest cell tractions on the substrate. A similar trend was observed when traction magnitude was normalized for cell size (Supplementary Fig. S7E) suggesting that increased traction magnitude on high substrate stiffness is not related to an increase in cell area. This data shows that vimentin is essential for stress fiber formation and the formation of larger focal adhesions and consequently increased traction force as a mechanoresponse to changes in the mechanical properties of the microenvironment.

**Figure 3:**
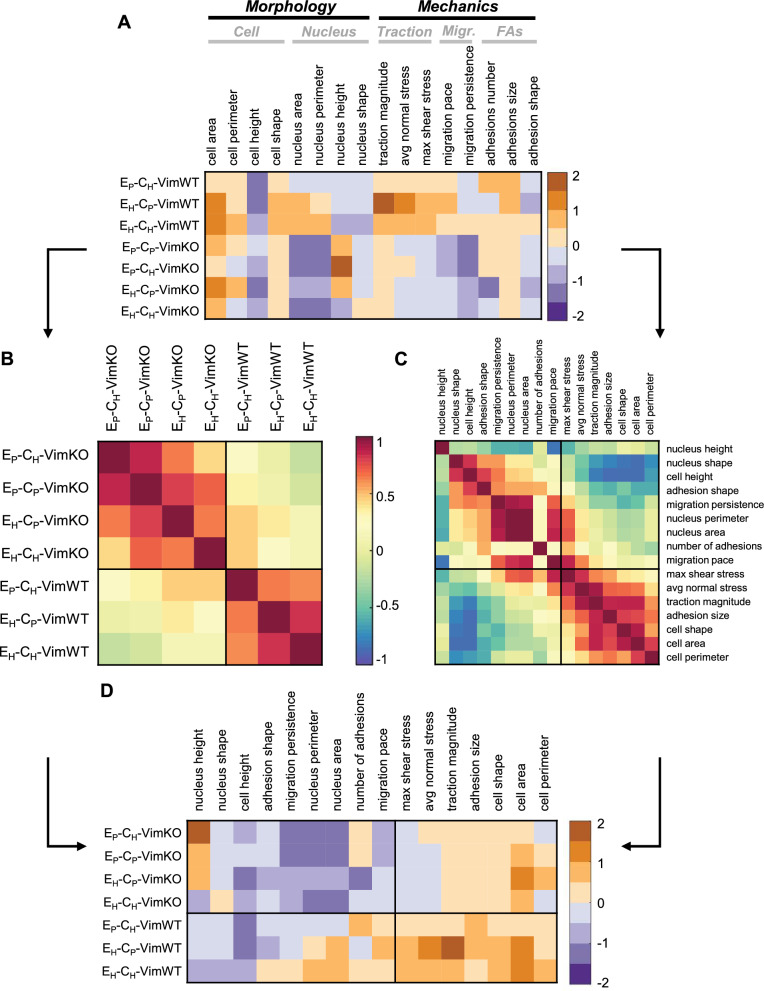
Vimentin depleted cells adopt differential morpho-mechanical phenotype after 24 h. (**A**) Matrix Z containing Z-Score of morpho-mechanical properties of different microenvironmental conditions with respect to control culture condition E_p_-C_p_-VimWT. (**B**,**C**) Ordered and clustered correlation matrices C and P between environmental conditions (**B**) and morpho-mechanical properties (**C**) respectively. (**D**) Ordered matrix Z based on clusters of conditions and properties correlation matrices. Additional information on the Z-score analysis including the unsupervised clustering algorithm can be found in the Supplementary Information.

The diversity of the morpho-mechanical response prompted us to systematically study whether the mechanical and morphological properties defining the cell’s response to microenvironmental changes were linked through universal relationships^35^. To that end, we identified a number of morpho-mechanical properties, averaged these properties over experimental repeats and categorized them into cellular morphology, nuclear morphology, cell mechanics, cell migration, and cell–matrix interactions. We computed a *m*x*n* matrix *Z* (Fig. 3A) in which each element represents the Z-score of a specific morpho-mechanical property of the cell as a response to a specific microenvironmental condition with respect to vimentin-expressing cells on a substrate having physiological stiffness and physiological ECM density. Thus, a positive or negative Z-score signifies that the mean of a specific physical property of a cell in a specific environmental condition is higher or lower compared to the control situation, respectively. Based on this *Z* matrix, we computed the cross-correlation matrices *C* (Fig. 3B) and *P* (Fig. 3C) which explored similarities between environmental conditions and morpho-mechanical properties respectively. A positive similarity between two environmental conditions signals that cells respond to these environmental conditions with similar morpho-mechanical properties, whereas a positive similarity between two morpho-mechanical properties signals that cells respond highly similar in terms of these properties in all environmental conditions. A negative similarity implies a substantial different response. Consequently, we used an unsupervised cluster algorithm^35,36^ to identify clusters, contoured in black, with highly correlated environmental conditions (Fig. 3B) and morpho-mechanical properties (Fig. 3C). We identified two clusters of highly correlated environmental conditions defined by vimentin expression (Fig. 3B). The unsupervised clustering algorithm detected two correlated clusters of morpho-mechanical properties (Fig. 3C). The first cluster demonstrated a high correlation between nuclear morphology and cell migration, which is in line with previous research^37^. Within the second cluster, we observed a high correlation between cellular morphology and cell mechanics. The clustering analysis of environmental conditions and morpho-mechanical properties was used to reorganize the initial matrix *Z* (Fig. 3D) and we observed that nuclear morphology and cell migration were negatively affected in VimKO cells while no strong effect was observed in VimWT cells. For cellular morphology and cell mechanics, VimWT displayed a strong increase in Z-score compared to VimKO cells independently of substrate. This highlights the differential morpho-mechanical state of vimentin-depleted cells in comparison to vimentin-expressing cells. Taken together, this analysis suggests that vimentin is required for cells to adapt to changes in the micromechanical environment within 24 h, a phenotype that is shared across cell types^19,21,38–41^. However, mechanoreciprocity occurs at different timescales^6^ which may result in cells being able to restore mechanical homeostasis on a variety of timescales. Thus, we wondered whether vimentin-expressing and vimentin-depleted cells had reached mechanical homeostasis (and thus if differences in their responses were permanent or only transitory).

### Vimentin is not necessary for establishing mechanical homeostasis

To test whether cells had reached a homeostatic morpho-mechanical state after 24 h, we cultured VimWT and VimKO cells on the different substrates (Fig. 2A) for 48 h and investigated the morphological and mechanical state of the cell. Specifically, we restricted our monitoring of the morpho-mechanical state of the cell at 48 h to cellular morphology and cell mechanics since these morpho-mechanical properties highly correlated in our similarity analysis at 24 h (Fig. 3C). For VimWT cells, cell area and cell traction forces at 24 h (Supplementary Fig. S8A,B) and 48 h (Supplementary Fig. S8C,D) varied across environmental conditions in a similar fashion indicating that VimWT cells have reached a mechanical homeostatic state already at this timepoint. Surprisingly, VimKO cells showed a similar mechanoresponse as VimWT cells at 48 h across microenvironmental conditions (Supplementary Fig. S8D) while VimKO cells did not adapt its traction forces to its microenvironment after 24 h (Supplementary Fig. S8B). This suggests that vimentin-depleted cells need time to respond and restore a mechanical homeostatic state. Nevertheless, VimKO cells cultured on physiological substrate stiffness showed decreased traction magnitude compared to VimWT cells while traction magnitude of VimKO cells on high substrate stiffness with physiological ECM density was comparable to that of VimWT cells (Supplementary Fig. S8D).

**Figure 4:**
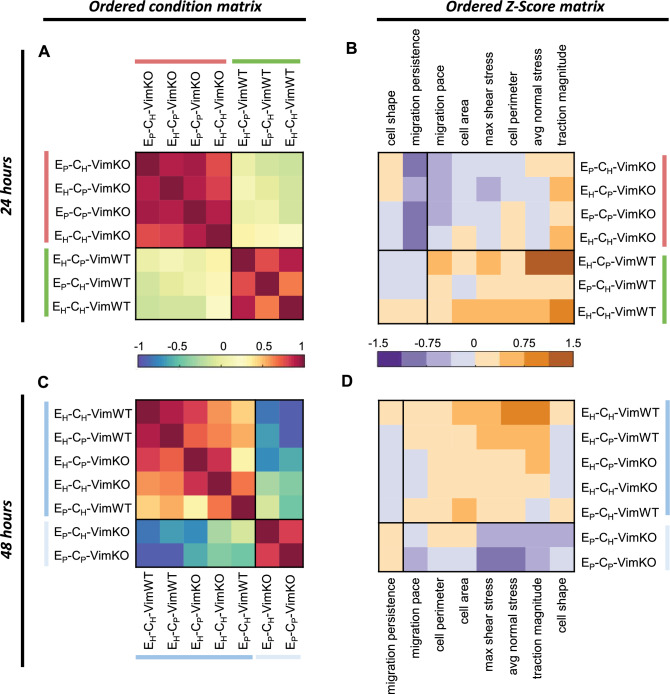
Increased substrate stiffness rescues loss of morpho-mechanical phenotype in vimentin depleted cells after 48 h. VimWT and VimKO cells were cultured for 24 h (**A**,**B**) or 48 h (**C**,**D**) on the different microenvironmental conditions. Ordered and clustered correlation matrices C between environmental conditions (**A**,**C**) and P between morpho-mechanical properties (data not shown) were produced and used to order matrix Z (**B**,**D**).

To corroborate the different response in time of VimKO cells, we repeated the unsupervised clustering analysis on experimental data sets collected at 24 and 48 h of cell culture. Cross-correlations of microenvironmental conditions and Z-score reorganization at 24 h shows that the presence of vimentin determines the morpho-mechanical phenotype of the cell (Fig. 4A,B). When culturing cells for 48 h on the substrates, the unsupervised cluster analysis again identified two clusters (Fig. 4C,D), these clusters presenting remarkable differences compared to two identified clusters at 24 h. The data at 48 h showed that VimKO cells can respond similarly to VimWT cells provided that the stiffness of the microenvironment is high enough, a feature not shown at 24 h of cell culture. These data highlight that vimentin is not required to establish mechanical homeostasis and indicate that there is a compensation mechanism of vimentin-depleted cells through modulation of matrix stiffness and time to establish a mechanical homeostatic state.

## Discussion

Intermediate filament vimentin is a structural protein within the cell and is essential for both cellular and mechanical integrity. Commonly reported phenotypes within (cells from) vimentin-depleted mice show alterations in cellular^15,19,40,42–47^ and nuclear^15,48–51^ morphology, adhesion to the microenvironment^19–21,23,40,42–44,52,53^, the ability to exert forces^16,21,34,40,45,47,54–58^, and deposition of ECM components^23,24,59–62^. In this systematic study, we showed that increased substrate stiffness compensated for increased ECM synthesis and crosslinking in vimentin-depleted cells (Fig. 1). Moreover, vimentin-depleted cells lost their ability to mechanically respond to changes in the microenvironment (Figs. 2 and 3), only to reacquire it at later stages if substrate stiffness is again increased (Fig. 4).

We systematically assessed the morpho-mechanical state of vimentin-expressing and vimentin-depleted MEFs by quantifying cellular and nuclear morphology, cell traction forces, cell migration and FA formation on collagen type I coated PAA hydrogel having different stiffness and ECM collagen densities. On substrates having physiological stiffness and ECM density, vimentin-expressing and vimentin-depleted cells behave similarly in terms of cellular and nuclear morphology, cell traction magnitude and FA size (Fig. 2). Differently from vimentin-depleted cells, changes in the mechanical properties of the microenvironment (stiffness and/or ECM density) in vimentin-expressing cells resulted in adaptation of their morpho-mechanical state. Vimentin-expressing cells presented an optimal response to substrate stiffness and ECM density in terms of larger FAs, higher tractions and increased cell spreading (Fig. 2). A cellular optimal response to changes in the microenvironment has been previously observed in several mechanobiological processes including cell spreading^28,29,63,64^, cell traction^64,65^, cell migration^63,64,66–68^, and YAP localization^69^. This optimal response may rely on the cell’s ability to form FAs, a dynamic process that is highly dependent on both substrate stiffness and ECM density and affects the mechanosensing capabilities of a cell. ECM density determines the number of FAs per cell^69^, while substrate stiffness in combination with the number of FAs determine the actomyosin contractility transmitted to the FAs, which in turn stimulates both growth and stabilization of these FAs^70^. FAs have been shown to stabilize faster on stiffer substrates due to a lower disassembly rate^71,72^. Upon increasing stress on FAs, cells form actin stress fibers that results in increased force loading of FAs—this process acts as a feedback loop in the growth and stabilization of FAs^72^. Through this loop, vimentin-expressing cells can sense and respond to changes in the mechanical properties of their microenvironment by adapting their morpho-mechanical state to restore mechanical homeostasis. Vimentin-depleted cells, instead, lose this mechanical property along with the IFs (Fig. 2).

Vimentin depletion alters FAs expression levels^23,40,43^, numbers^21,42,44^, size^20,21,43,44,52,53^, and dynamics^19–21,44,52,53^ hinting at a regulatory role for vimentin in the formation of FAs and, thus, the mechanosensing capabilities of a cell. Additionally, Ostrowska-Podhorodecka and colleagues have proposed that vimentin acts as an adaptor protein for FA proteins^43^. In this study, we observed no changes in FA size, impaired stress fiber formation, and no response in cell traction force in vimentin-depleted cells upon mechanical changes in the microenvironment (Fig. 2) suggesting that the early morpho-mechanical phenotype of MEF cells is determined by vimentin expression (Fig. 3). Vimentin-expressing cells reach a mechanical homeostatic state earlier (24 h) than vimentin-depleted cells and show no further changes in their morpho-mechanical features with time (Fig. 4). Instead, vimentin-depleted cells reach this state at a longer timescale (48 h). Vimentin-depleted cells cultured on a stiff substrate independently of ECM density for 48 h adopt a phenotype comparable to that of vimentin-expressing cells independently of substrate condition (Fig. 4).

Adaptation of the morphological and mechanical state of a cell occurs at a timescale of seconds to hours^73–77^, which is in line with the observation that vimentin-expressing cells have reached a homeostatic state after 24 h. However, vimentin-depleted cells reach mechanical homeostasis only after 48 h, therefore, pointing towards the existence of a compensation mechanism that occurs in a timespan of days. It is worth noting that production and remodeling of ECM is a process occurring at longer timescales. Previous research has provided evidence for a regulating role of vimentin in stabilization of collagen mRNAs^59,60^, collagen fiber alignment^62^ and ECM production and remodeling^23,24^. Our data on physiological substrate stiffness show that vimentin depletion results in increased ECM synthesis and collagen crosslinking (Fig. 1), processes that have been correlated with a local increase in environmental stiffness^78,79^. Also, culturing vimentin-depleted cells on stiffer substrates decreases ECM production and collagen crosslinking in comparison to physiological substrate stiffness (Fig. 1).

Concluding, we have further elucidated the role that vimentin plays in mediating processes of cellular mechanoreciprocity and, consequently, mechanical homeostasis. Indeed, while the restoration of mechanical homeostasis for cells is not hindered on longer timescales, IF vimentin provides cells with a fast mechanical adaptive response to changes in the mechanical properties of the microenvironment. In that respect, our results point towards the existence of a cellular mechanism that enables vimentin-depleted cells to compensate their loss of vimentin in restoring mechanical homeostasis, even if only on a slower timescale. We speculate that this compensatory mechanism consists in vimentin-depleted cells increasing the production of collagen and collagen crosslinkers (as observed in Fig. 1). Increased collagen crosslinking results in an increase in matrix stiffness^78–80^ and therefore alterations of the mechanical properties of the matrix. This is consistent with observations from previous in vivo studies where it was shown that vimentin-depletion leads to arterial stiffening^23,24^. With time, this compensatory matrix stiffening may allow vimentin-depleted cells to restore the homeostatic morpho-mechanical phenotype characteristic of vimentin-expressing cells (as observed Fig. 4). This novel insight and theory, albeit not fully mechanistically proven, advances the understanding of the regulatory role of vimentin in mechanoreciprocity and mechanical homeostasis.

## Materials and methods

### Cell culture

Vimentin wildtype (VimWT) and vimentin knockout (VimKO) mouse embryonic fibroblasts (MEFs) were kindly provided by John Eriksson (Åbo Akademi, Finland).

### Polyacrylamide (PAA) substrate preparation

PAA gels with a Young’s modulus of 12 kPa (E_P_) and 27 kPa (E_H_) were prepared on glass bottom well plates (MatTek/CellVis) of microscope glass slides. PAA gels were functionalized using 1.0 mg/ml Sulfo-SANPAH (Pierce) and coated with 25 µg/ml (C_P_) or 100 µg/ml (C_H_) rat tail collagen type I (Corning).

### Immunofluorescence staining

MEFs were fixed in paraformaldehyde (ThermoFisher), permeabilized using Triton-X-100 (Sigma) and blocked in bovine serum albumin (Roche). Primary antibodies used in this study: Collagen type III (ab7778, abcam), Paxillin (ab32084, abcam), phosphorylated myosin light chain (3675S, Cell Signaling) and Vimentin (ab20346, abcam). F-actin, collagen and the nucleus were stained using Phalloidin (Sigma), CNA probe (CNA35-OG488) and DAPI (Merck) respectively. Images were acquired using an epifluorescence (Leica DMi8) or confocal microscope (Leica SP8X).

### Gene expression analysis

RNA was isolated using a RNeasy kit (Qiagen). Quantitative real-time polymerase chain reaction (qPCR) was performed on complementary DNA and quantified using the Pfaffl method.

### Traction force microscopy

Cells and fluorescent beads were imaged overnight. The reference image was obtained after removal of the cells. Timelapse images were aligned and cropped according to the reference image. Bead displacements were computed using Particle Image Velocimetry. Cell tractions were computed by Fourier transform-based traction microscopy. Traction magnitude was calculated as median value over space (the cell) followed by mean value over time.

### Z-score analysis

The Z-score is a measure of a morpho-mechanical property $x$ as a result of a specific environmental condition. The Z-score is defined as $z=(\overline{x }-\overline{{x }_{c})}/{\sigma }_{c}$ in which $\overline{x }$ is the mean of morpho-mechanical property $x$ within a specific culture condition, $\overline{{x }_{c}}$ is the mean of physical property $x$ of the control culture condition (E_P_-C_P_-WT) and ${\sigma }_{c}$ is the standard deviation of physical property $x$ of the control culture condition. Correlations between morpho-mechanical properties and culture conditions were calculated using cosine similarity analysis. An unsupervised clustering algorithm was used to detect clusters in the Z-score matrix.

### Statistics

Data is presented as mean ± standard error of the mean (SEM). Normality was tested using Shapiro Wilk test. Statistical tests used in this study were an unpaired student t-test (gene/protein expression analysis), a Mann–Whitney test and a Kruskall-Wallis test followed by Dunn’s multiple comparison test (quantification of morpho-mechanical state).

See additional details in SI Appendix, SI Materials and Methods.

### Data and software availability

Software for the analysis of FAs (SFAlab)^81^ is publicly available through this link. Software for the traction force microscopy analysis is available on request. Software for cosine similarity analysis and unsupervised clustering is publicly available through this link. All study data is available and can be obtained from a DOI-minting repository^82^. The raw data is available upon reasonable request. Data regarding ECM can be requested to C.M.S. while data and processing codes regarding the morpho-mechanical state of the cell and Z-Score input can be requested to V.C.

### Supplementary Information

Supplementary Information.
